# What makes mental health and substance use services youth friendly? A scoping review of literature

**DOI:** 10.1186/s12913-019-4066-5

**Published:** 2019-04-27

**Authors:** Lisa D. Hawke, Kamna Mehra, Cara Settipani, Jaqueline Relihan, Karleigh Darnay, Gloria Chaim, Joanna Henderson

**Affiliations:** 1 Centre for Addiction and Mental Health, 80 Workman Way, Toronto, ON M6J 1H4 Canada; 2 University of Toronto, 250 College Street, Toronto, ON M5T 1R8 Canada

**Keywords:** Youth friendliness, Mental health services, Substance use services, Youth engagement

## Abstract

**Background:**

There are increasing calls to make mental health and substance use services youth friendly, with hopes of improving service uptake, engagement and satisfaction. However, youth-friendliness in this area has not been clearly defined and there is a lack of information about the characteristics that make such services youth friendly. The purpose of this scoping review was to examine the literature available on youth-friendly mental health and substance use services in order to identify the characteristics, outline the expected impacts, and establish a definition.

**Methods:**

A scoping review of seven databases and grey literature sources was conducted. Twenty-eight documents were retained as relevant to the research questions. Relevant data from these documents was extracted, analyzed and presented to stakeholders, including youth, caregivers and service providers to validate and refine the results.

**Results:**

Youth-friendly mental health and substance use services include integrated, inclusive, confidential and safe organization and policy characteristics; bright, comfortable, environment with informational materials; welcoming and genuine service providers with appropriate communication and counselling skills; an accessible location; minimal wait times; and individualized and innovative approaches. All areas in which youth friendliness should be implemented in a mental health and substance use service organization had a core value of youth voice.

**Conclusion:**

Improving the youth friendliness of mental health and substance use services includes incorporating youth voice in organization, policy, environment, service providers, and treatment services, and has implications for treatment uptake, engagement and satisfaction. Further research is required to determine the impact of youth friendliness in such services.

**Electronic supplementary material:**

The online version of this article (10.1186/s12913-019-4066-5) contains supplementary material, which is available to authorized users.

## Background

The United Nations has defined ‘youth’ as a period of development between the ages of 15 to 24 years [1]. This is a crucial period for the emergence of mental health challenges, with some 70% of mental health disorders arising before adulthood [2]. An epidemiological study suggests that mental health and substance use (MHSU) disorders occur in some 12.6% of people under 18 years of age in Canada [3]. In addition, Statistics Canada data shows that suicide is the second most common cause of death among youth [4], making youth-specific MHSU services a critical area for development.

Despite treatments being available, many mental health disorders in youth remain untreated [5]. Barriers to treatment in this age group include inadequate awareness of mental illness and the treatments available, youth preference for self-management, stigma, lack of screening and identification, treatment access issues, system fragmentation, and a lack of youth-specific evidence-based treatments [6–12].

In order to improve service utilization, there is increased attention to factors affecting youth engagement in services [13, 14], including the establishment of youth-friendly mental health services. The World Health Organization (WHO) developed a framework for youth-friendly health services in general; their recommendations include access to acceptable health services, and the right services for each individual, provided in an appropriate way [15]. WHO proposes that making health services youth friendly may improve service utilization, with increased youth engagement and satisfaction. However, this framework was developed with an overall health perspective, including the full range of physical health services, i.e., it is not specific to MHSU services. These recommendations may or may not be relevant to MHSU services.

Although some literature has addressed youth-friendly MHSU services, there is a lack of clarity about the definition and characteristics of youth friendliness in MHSU service contexts. Without an understanding of how the youth friendliness of MHSU services is defined and how it is characterized, it is difficult to establish the extent to which services are youth friendly and identify areas for improvement.

The objective of this scoping review is to examine the literature available on youth-friendly MHSU services from the perspectives of youth, caregivers, and service providers. The main focus is to identify the characteristics of youth friendliness in MHSU service settings, formulate a definition, and outline the expected impacts.

## Methods

For this scoping review, we have accepted the United Nations definition of youth (i.e. age 15–24 years) [1], but allowed for flexibility in the defined age range based on definitions used in the literature. The methodology is described in detail in Hawke et al. [16]. Our methodological approach was based on established guidelines [17–20] and included six steps:

### Defining the research question

This scoping review seeks to answer the following research questions:

- What are the characteristics of youth-friendly mental health and substance use services?
- What is the expected impact of youth-friendly mental health and substance use services on service uptake, engagement and satisfaction?
- How are youth-friendly mental health and substance use services defined?

### Identifying relevant studies

A search of seven electronic databases (Table 1) was conducted to identify literature produced over the last fifteen years (2002–2017), with an extensive grey literature search based on the Grey Matters research tool [21]. The database search was developed in Medline using text word and subject heading terms for the particular concepts of ‘youth friendliness’ and adolescent or youth, mental health or substance use (see Additional file 1). This search was then adapted to other databases. Since ‘youth friendliness’ is not a specific subject heading, and we wanted to explicitly address this concept (e.g., we were not interested in the broad concept of ‘youth appropriateness’), we searched for citations that included either the word ‘friendly’ or ‘welcoming.’ Google Advanced Search was used to conduct a wider search for grey literature. We also identified additional literature from the reference lists of relevant documents.

**Table 1:** List of Databases Searched for the Review

No	Name of database
1	Medline
2	PsycINFO
3	CINAHL
4	EMBASE
5	Cochrane Library
6	Applied Social Science Index and Abstracts
7	Campbell Collaboration Library

### Study selection

**Fig. 1:**
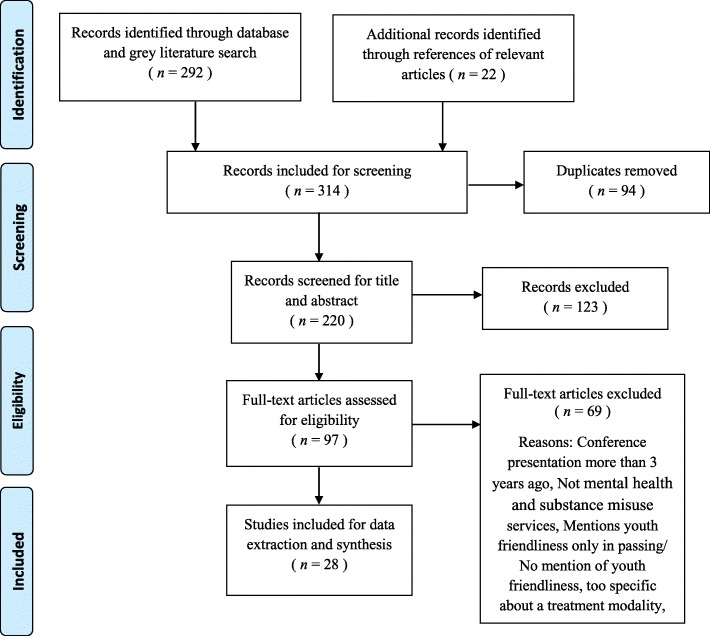
Flowchart of the Documents Included, Based on PRISMA Guidelines

A total of 292 documents were identified through the database and grey literature search process, and 22 documents were added from reference lists of relevant documents. Ninety-four duplicate studies were removed and 220 studies were screened on the basis of inclusion and exclusion criteria (Table 2) at the title and abstract level. The Cochrane-recommended software program, Covidence [22], was used for title and abstract screening. Two raters independently screened and rated documents for relevance; any discrepancies were resolved by a third independent rater. One hundred and twenty-three studies were excluded at this stage; the remaining 97 studies were screened at the full text level, of which 28 were selected for final inclusion in the review (Fig. 1).

**Table 2:** Inclusion and Exclusion Criteria

Inclusion Criteria	Exclusion Criteria
• Research and non-research studies	• Documents addressing adults only or children only
• Documents relevant to any mental health and substance use service setting (i.e., community centers, hospitals, primary care)	• Documents not discussing services for mental or behavioral health or substance use (e.g., focus on physical health services)
• Documents focusing on adolescents, youth, young adults, or emerging adults	• Documents mentioning youth friendliness only in passing/not explaining youth friendliness in ways that answer the research question
• Documents irrespective of gender/sex, and ethnicity	• Documents specific to a particular treatment modality rather than the service setting as a whole (e.g., youth adaptations of cognitive-behavioral therapy)
• Only documents addressing mental/ behavioral health or addiction service settings	
• Only documents specifically discussing the definition, characteristics, or expected impact of youth friendliness in these settings	• Conference presentation more than 3 years ago
	• Documents originating from developing countries, where youth culture may not be generalizable to that in developed countries

### Data extraction

Data from the final 28 documents were extracted using a data extraction form by two research assistants and verified independently by a third researcher. For further details about the data extraction form, see the published study protocol [16].

### Collecting, summarizing and reporting of data

The collected data were summarized descriptively; the characteristics of youth-friendliness were found to fall into four overarching spheres: organization and policy characteristics, environment characteristics, service provider characteristics, and treatment/service characteristics. Additional data was extracted to describe the anticipated impacts of youth friendliness. The sum of the findings was used to formulate a definition of youth-friendly MHSU services.

### Stakeholder consultations

In order to validate the results, focus groups were conducted with 32 stakeholders, including youth, caregivers and service providers. We consulted standing advisory groups of service providers, caregivers and youth; since attendance at these groups is variable, our consultations included 19 service providers, 8 caregivers and 5 youth. Service providers were 13 female, 3 male, and 2 trans/non-binary participants, with an average age of 37.8 (SD = 11.4); a majority (66.6%) were Caucasian. Caregivers were 7 female and 1 male participants, with an average age of 50.9 (SD = 8.8); a majority (75.0%) were Caucasian. The mean age of youth was 20 years (range 18–23 years), with 4 female and one male participant, and two (40.0%) identifying as Caucasian. Ethics approval for stakeholder consultations was obtained from the Centre for Addiction and Mental Health Research Ethics Board. Participants were asked to provide feedback about the preliminary results of the review and to identify any missing components. They were asked “What part of this information makes sense?”, “What part of this information does not make sense?” and “What information would you add to these findings?” Their feedback was used to refine the results.

## Results

Out of the 28 papers included in the review, 18 were peer-reviewed journal articles and 10 were grey literature documents; the 28 documents originated from 6 countries, representing 19 research teams. They presented the perspectives of service-seeking youth, caregivers and service providers. Results are summarized in Table 3. Data from the retained documents were found to fall into four main aspects of services: organizational/policy characteristics, environment characteristics, staff characteristics, and treatment/service characteristics; additional data suggested the impacts that youth-friendly MHSU services might have and proposed some aspects of a definition. Stakeholders provided feedback in focus groups, which was incorporated into the results.

**Table 3:** Overview of Documents Obtained in this Scoping Review

Author (Reference Number)	Location	Type of document	Perspectives/ Description of services	Organization and policy characteristics	Environment characteristics	Staff characteristics	Treatment/ Service characteristics	Expected Impact	Definition
Armstrong [23]	Canada	Peer reviewed journal article	Youth and Staff perspectives	Youth voice	–	–	–	↑Youth engagement may lead to ↓stigma, ↓suicidality, ↑coping, ↑youth connections	Youth participation in service design and delivery, under close support of professionals
Bardash [24]	USA	Dissertation	Youth and Staff perspectives	Transitional age focus, terminology	Physical layout and décor, Informational materials	–	Location and access,	Youth-friendly environment may lead to ↑service uptake	–
Boyden, Muniz, & Laxton-Kane [25]	UK	Peer reviewed journal article	Youth perspectives	–	–	Communication and counselling skills	–	–	–
Davidson et al., [26]	Canada	Peer reviewed journal article	Description of services	–	–	–	–	–	Knowledge of, appreciation for, and lack of judgment towards youth culture
Davidson et al.[27]	Canada	Peer reviewed journal article	Youth perspectives	–	–	–	–	–	Knowledge of, appreciation for, and lack of judgment towards youth culture
Dixon et al. [28]	Australia	Peer reviewed journal article	Staff perspectives	–	–	Communication and counselling skills	–	–	–
Goodwin [29]	New Zealand	Presentation	Description of services	Youth voice, Integrated services, Confidentiality, Inclusive and culturally diverse	–	Youth voice, Paradigms of working with youth	Appointment times, Cost, First contact and assessment,	–	Youth accessibility, appropriateness, affordability, and confidentiality
Hyman et al. [30]	Canada	Peer reviewed journal article	Youth perspectives	–	–	Values and attitudes	–	Youth-friendly service provider may lead to ↑treatment engagement	–
James [31]	Australia	Peer reviewed journal article	Description of services	Youth voice	–	–	–	–	Listen to youth, do so willingly and frequently, and take notice of what it hears
Kiselica [32]	USA	Peer reviewed journal article	Description of services	Inclusive services	Physical layout and décor	Paradigms of working with youth, Communication and counselling skills	Appointment times, Recreational activities, Group activities, Innovative services	Youth-friendly service provider and recreational activities may lead to ↑ treatment engagement	Wide variety of strategies that appeal to youth and that facilitate establishment and maintenance of rapport
Kiselica [33]	USA	Book chapter	Description of services	–	–	Paradigms of working with youth	–	–	Informal settings, flexible time schedules, instrumental activities, humor, self-disclosure, psychoeducational groups
Kiselica and Englar-Carlson [34]	USA	Book chapter	Description of services	–	Physical layout and décor	Paradigms of working with youth, Communication and counselling skills,	Appointment times, Recreational activities, Group activities, Innovative services	Youth-friendly service provider may lead to ↑ treatment engagement	Wide variety of strategies that appeal to youth and that facilitate establishment and maintenance of rapport
McCann and Lubman [35]	Australia	Peer reviewed journal article	Youth perspectives	–	–	Values and attitudes	Appointment times, Youth practitioner fit, Individualized response	Youth-friendly service provider may lead to ↑service satisfaction	Youth feel engaged, valued, respected, supported to take control of their lives
McGorry [36]	Australia	Peer reviewed journal article	Description of services	–	–	–	–	–	Services that acknowledge and respond to cultural and developmental issues
Medlow et al. [37]	Australia	Peer reviewed journal article	Youth perspectives	–	Physical layout and décor	Welcoming staff	–	–	Greater consumer and carer involvement in planning and delivery
Muir, Powell & McDermott [38]	Australia	Peer reviewed journal article	Youth perspectives	Integrated services, Confidentiality, Transitional age focus	Physical layout and décor	Welcoming staff, Values and attitudes	Youth voice, Location and access, Appointment times, Cost, Youth Practitioner fit	Youth-friendly services (e.g. text messages) may lead to ↑treatment engagement	WHO youth-friendly services definition
Paul Hamlyn Foundation and Mental Health Foundation [39]	UK	Guidelines/ Framework	Description of services	Youth voice, Confidentiality, Appropriate promotional approaches, Technological platforms,	–	Youth voice	Location and access, Appointment times, First contact and assessment, Youth practitioner fit, Recreational activities	Youth engagement may lead to ↑service uptake, improved mental health, ↑youth empowerment, ↑transparency, ↓stigma, and ↑career development among youth	WHO ‘You’re Welcome’ criteria
Persson, Hagquist & Michelson [40]	Sweden	Peer reviewed journal article	Youth perspectives	Safe space	Youth voice, Physical layout and décor	Welcoming staff, Communication and counselling skills	Location and access, Appointment times, Caregiver involvement, Recreational approaches, Innovative approaches	–	–
Pinto [41]	UK	Peer reviewed journal article	Description of services	–	–	–	–	–	Adapt frameworks to meet current situation
Pope [42]	UK	Peer reviewed journal article	Youth perspectives	Appropriate promotional approaches	–	Youth voice, Young staff members	–	–	Younger staff, ↑visibility, youth designed publicity
Rhodes [43]	USA	Dissertation	Staff perspectives	–	–	–	Innovative approaches	–	–
Rickwood et al. [44]	Australia	Guidelines/ Framework	Youth, Caregiver, Staff perspectives	Youth voice, Integrated services, Inclusive and culturally diverse services	Physical layout and décor, Informational materials	Welcoming staff, Values and attitudes, Communication and counselling skills	Individualized response	–	WHO youth-friendly services definition
Rickwood, Van Dyke & Telford [45]	Australia	Peer reviewed journal article	Description of services	–	Physical layout and décor, Informational materials	–	–	–	Youth participation in own health care and well-being management
Rosen and Howe [46]	Australia	Presentation	Description of services	Confidentiality, Inclusive services	–		Location and access, Appointment times, Cost	–	–
Scheve, Perkins & Mincemoyer [47]	USA	Peer reviewed journal article	Youth perspectives	Youth voice	–	–	–	Listening to youth and applying their ideas may lead to ↑youth engagement in services	–
Stromback, Malmgren-Olsson & Wiklund [48]	Sweden	Peer reviewed journal article	Youth perspectives	–	–	–	–	–	WHO youth-friendly services definition
Ontario Centre of Excellence for Child and Youth Mental Health [49]	Canada	Guidelines/ Framework	Description of services	Confidentiality, Safe space, Inclusive and diverse	Youth voice, Physical layout and décor	Communication and counselling skills	Location and access, Appointment times, Group activities	–	Safe space – where one feels respected, valued, can express themselves authentically without fear of being judged
Youthline [50]	New Zealand	Guidelines/ Framework	Description of services	Appropriate promotional approaches, Technological platforms	Informational materials	**–**	**–**	**–**	Defined youth-friendly mental health resources

### Organization and policy characteristics

In order to truly make MHSU services youth friendly, findings suggest that substantial system level changes may be needed^**†**^. This may require incorporating the following characteristics into the organization and policies of an MHSU service and more broadly into the MHSU service system as a whole:

* = Supported/expanded upon by youth consulted.

^#^ = Supported/expanded upon by caregivers consulted.

^†^ = Supported/expanded upon by service providers consulted.

#### Youth voice: youth engagement at the organization/policy level

Youth are considered to be best positioned to judge the youth friendliness of MHSU services [31]. A number of authors therefore suggested that youth should be engaged in organization and policy development, implementation and evaluation of services [23, 29, 44], through youth advisory or consultation groups [29, 44]. Tokenistic youth engagement needs to be avoided^†^, and youth engagement should be ongoing; an example includes involving youth on the board of directors^#^. This may support the appraisal and accountability of the service [29]. Diverse youth from a wide variety of locally-relevant cultures and identities should be engaged^#^. Youth expect organizations to work ‘with youth’ (i.e., alongside youth) rather than ‘for’ or about youth, which they feel helps youth retain their agency and power*. Youth engagement helps youth develop skills that they can use in turn to provide input into services [29] and to help the organizations become more youth friendly. Several papers provided recommendations on engaging youth effectively in service development/planning [29, 31, 39, 47]. An important aspect includes providing youth with incentives that are practically appealing, to help them engage at the organizational level [29]^†,#,^*. This may help youth take the first step to enter a service organization, which may then lead in turn to valuable youth engagement at both the organizational and service levels*.

#### One-stop shop/integrated services

In order to be youth friendly, the literature posits that youth-serving agencies should, at the organizational/policy level, offer comprehensive co-located services for mental and physical health, substance use, and social and vocational support [38, 44]. This may also include housing services^†^. Integrated services are believed to make youth feel safe, since they can meet various practitioners to address a wide range of needs in an environment to which they are accustomed [38]. Co-locating services prevents youth from spending time or money to access diverse services in different locations*; in addition, they can receive services without it being obvious to their peers what type of services they are seeking. The ‘one-stop-shop’ approach can also prevent youth from having to repeat their stories multiple times [38]; in an integrated service environment, organizations may share their databases about the youth’s history^#^ or give the youth access to their file to share, further preventing the repeated telling of their stories^#^. An integrated service experience can be supported by providing a wraparound worker who can help youth access multiple services^#^. Goodwin [29] recommended embedding youth friendliness across partnering organizations, i.e., the youth friendliness of the main youth-serving agency is insufficient if the collaborating partners do not also uphold youth-friendly approaches.

#### Confidentiality and privacy

Confidentiality and privacy are critical to youth-friendly MHSU services [29, 38, 39, 49]. Thus, organizations should have clear policies about confidentiality, rights and responsibilities, and consent to involve others in treatment [46]. Although integrated services can prevent youth from having to retell their stories, youth also appreciate confidentiality*, i.e., they only want their information shared with their consent, even among colleagues who are directly serving the youth*. Youth should therefore be informed about what is confidential and what is not, including confidentiality vs. sharing with parents and the limits to confidentiality [38]. Youth’s explicit permission should be obtained before releasing information to parents [38].

#### Appropriate promotional approaches

Often, youth may not be aware of the existing MHSU services available to them [39], which is an access barrier at the organizational level. Thus, a youth-friendly service is one that invests appropriate resources to promote services, using youth-informed methods to reach the target youth [39]. Diverse youth should be engaged in developing youth-friendly promotional materials and strategies [42, 50]. Youth engagement in the promotion process is key as youth can explain services in youth-friendly terms and dispel myths [42]. It may be important to include information about youth engagement in promotional materials, including the incentives being provided to youth^†,#^. Knowing that engagement and incentives are available may motivate youth to take the first step in engaging with the organization*^,†,#^. Youth also suggest displaying posters about services in spaces that youth frequently visit*. Peer mentors may be engaged to promote services in schools and educate youth about mental health issues^#^. To promote MHSU services specifically to male youth who may be more difficult to reach, it may be helpful to involve male counsellors or youth in targeted promotional activities to increase visibility of male-role models [42].

#### Technological platforms

Since today’s youth are avid users of technology, MHSU service organizations should make the commitment at the organizational level to leverage technological platforms to reach youth for promotional, informational, and psychoeducational activities^†^. For example, social media platforms/websites can be leveraged [39, 50]. The type of social media used to promote services may affect the level of youth engagement and service seeking. However, it should be kept in mind that youth may not follow certain social media pages if they are concerned about being stigmatized by their peers*; technological platforms used to promotes services should have generic names or icons that enable youth to use them discreetly and should be private and confidential [50]. Websites should provide clear information that help youth identify issues, without providing diagnoses; they should also offer practical advice on how to stay mentally healthy and provide links to multiple resources/available services [50].

#### ‘Safe’ space versus ‘brave’ space

Youth-serving agencies should commit at the policy level to making their service setting a safe space in which youth feel respected and valued, without being judged on any grounds [49]. Youth appreciate interpersonal interactions that make them feel safe and welcome in a MHSU service [40]. In order to provide a safe space, the key values of the MHSU service need to be identified and embedded in organizational policy. Safe-space values include no discrimination (e.g., discrimination against immigrant youth, racialized youth etc.)*, using a trauma-informed lens*, having a conflict resolution policy, placing safety first, and having back up clinical support [49], as well as understanding intersectionality*^,†^ and establishing an anti-oppression policy^†^. The youth consulted noted that it may be impossible to ensure that a space feels safe to all service users, as the types of discussions and interactions taking place in the space cannot be completely controlled and some of the topics that youth wish to discuss may not feel safe to them (e.g., discussing traumatic experiences)*. Thus, youth suggest that it would be more youth friendly to establish the service setting as a ‘brave space’ rather than a ‘safe space’, since youth are being brave by discussing sensitive issues*.

#### Transitional age focus

To be youth friendly, organizations need to recognize that ‘youth’ is a transitional age that is distinct from childhood and adulthood, with developmental implications. Youth may not feel comfortable in services that are targeted towards adults or children [24]. However, the 12–25 age range [38] is also not a homogenous age group; services should cater to varying ages and developmental stages, which is a balancing task that can be difficult to achieve, but should be prioritized in the context of youth-friendly MHSU services [24, 38]. The youth consulted were particularly interested in ensuring smooth transitions between services; for example they appreciate having guidance in the transition from children’s services to youth services or from youth services to adult services*.

#### Inclusive and culturally diverse services

A MHSU service may be perceived as more youth friendly when it is accessible for youth with a diversity of needs and abilities and respects their cultural background [44, 49]. In order to build trust with youth, organizations need to develop policies regarding knowledge of youth cultures, including sex, gender, race, discrimination, etc., and train staff to discuss such sensitive issues [32, 46]. For example, youth belonging to the LGBTQ community should be able to talk about their issues openly, without stigma [44]. Similarly, Indigenous youth may appreciate groups focusing on Indigenous issues, without excluding other youth [44]. For example, Goodwin [29] established a bicultural service, taking into consideration the Western-European and Maori cultures reflecting the local cultural context. Although cultural competency (i.e., knowledge about common experiences of different cultural populations) may be an essential component of youth-friendly services, youth stressed that training staff in cultural competency is not equivalent to having staff with direct lived experience of the culture in question*. Thus, organizations striving to be youth-friendly should employ service providers from diverse groups, with a variety of cultural backgrounds.

#### Terminology

Bardash [24] reported that youth find the current terms used in MHSU services to be pathologizing or disease-based, and suggested that more youth-friendly terms should be used, for example changing the term therapist to counsellor. There may be a need to develop terms or labels that youth and caregivers are comfortable using^†^. Similarly, caregivers noted that labelling youth with a diagnosis may create stigma and be harmful for some youth, but that, for other youth, having a diagnosis may help them understand their issues and access appropriate services^#^. Thus, using terminology that is not stigmatizing, yet helps youth access the services they need, may make services more youth friendly.

### Environment characteristics

One aspect that draws youth with MHSU challenges to the service setting may be its environment. Many characteristics of the environment that contribute to making a MHSU service setting youth friendly were highlighted in both the literature and stakeholder consultations.

#### Youth voice: youth engagement in environment development

Youth should be provided with opportunities to make the service environment their own [49]. For example, youth engagement teams can be engaged in the design of the space, such as choosing the color of the walls or making murals^†^. Service-seeking youth may also appreciate influencing their service environment, e.g., deciding to have music in the background during a session [40].

#### Physical layout and décor

Youth prefer a comfortable, relaxed, appealing, and welcoming physical environment [24, 44, 49]. They appreciate a non-clinical atmosphere, i.e., avoiding the white walls one might expect in a hospital setting [24, 40, 44]. There should be windows in the space^#^, and it should be brightly illuminated*. Youth have reported preferring bright colors and comfortable furniture like couches, which makes the space more informal and comfortable [37, 38, 40, 44]. Colorful artwork and posters of modern music and media personalities also create a vibrant atmosphere [24, 44]. The artwork and posters should reflect diversity (e.g., displaying gay/transsexual couples or people from diverse ethnical or cultural backgrounds) and be in tune with the culture of the youth served^#^. Youth might be invited to display their own artwork in the center [45]. Although the literature suggested including magazines in the space to make the environment youth friendly [32, 34], this may now be an outdated recommendation, as stakeholders mentioned that youth currently prefer using social media*^,#,†^. Some youth may appreciate adult coloring books* or “Zines” (i.e., youth-published work)^†^. Similarly, music in the waiting room is considered to make the environment youth friendly [24, 40], since it relaxes youth and puts them at ease*, but the choice of music may be different for different youth*^,#,†^. While designing a space, it is important to find a balance between a professional versus youth-oriented decor, since some youth may not appreciate services aimed excessively towards youth [38]^†,#^.

There should be a quiet area in the setting for youth to de-stress [49]; youth added that there should be a space to do school work while waiting for appointments*. Caregivers further suggested a smoking area for youth, as some youth may use smoking to manage their emotions^#^. The waiting area can be enhanced by providing easy access to condoms, dental dams^#^, and harm reduction supplies^†^. Youth may want access to electronic entertainment, e.g., computers and iPads with internet access in the common waiting area [38, 44]. All three consultation groups expanded on this notion, noting that Internet access (Wi-Fi) has become an essential part of youth-friendly MHSU services*^,#,†^; youth mentioned feeling less anxious when Wi-Fi is available to occupy themselves while waiting for an appointment, search for MHSU concepts, communicate with friends or family, or search for public transit*.

Session rooms should also be designed with youth friendliness in mind. For example, they might have refreshments available [40], which are particularly appreciated by youth*^,†,#^. In addition, youth appreciate having small tactile objects available to keep their hands busy (“fidget toys”), as well as other features such as musical instruments or sports collectibles to stimulate conversation [32, 34]. This may help youth relax and communicate more easily*.

#### Informational materials

Several papers suggested that youth appreciate having informational brochures available in the waiting area [24, 44, 45]. Youth-friendly brochures may provide information about a range of topics, in plain language^†^ and in multiple languages, including content in line with best practice guidelines; information should be up-to-date and strengths based, inspiring hope and normalizing help seeking [50]. It may be appreciated if such information is displayed in plain sight along with a range of other information, where youth feel they can access the information discreetly [50]. Since youth may not always read brochures,^†^ this information should also be available through other channels (e.g., websites, social media) [50].

### Staff and service provider characteristics

It has been suggested that youth find the youth friendliness of a service provider potentially more critical than their qualifications [44]. The characteristics of a youth-friendly service provider are described below.

#### Paradigms of working with youth

The literature highlights that staff paradigms (i.e., ways of viewing the world and its various problems) about working with youth are crucial for the development of services relevant to youth [29]. Staff are encouraged to challenge their pre-existing, longstanding ways of working in the mental health system [29]. Similarly, service providers may abandon the professional constraints of conventional counselling settings and alter the service settings for youth depending on what they prefer, e.g., doing a project or walking in the park together, etc. [32, 34]. Challenges for service providers may include conquering their own biases (e.g., gender-related biases) and clinical training and beliefs (e.g., maintaining professional distance to preserve objectivity) [32–34].

#### Youth voice: youth as service providers

The youth voice can be an integral part of the interprofessional team in the form of peer counselors to enhance youth friendliness. Since many youth like to talk to and listen to other youth, youth may be engaged in helping each other during times of stress [42]. Youth may also express an interest in learning counselling skills and becoming peer counsellors themselves [42]. The ‘Right Here’ project found that when peer counsellors are engaged, this provides them with an opportunity to share their lived experience, reduces stigma and decreases isolation [39]. All three stakeholder groups suggested benefits of having young peer workers at the front door, for example a peer greeter*^,†,#^ who can welcome them and guide them through the site’s offerings to help to make them feel comfortable.

#### Young staff members

While youth engagement mechanisms such as youth advisory groups and peer counselors are critical, employing young staff members, such as young clinicians, may also add to youth friendliness. Some youth may relate better to younger staff members who identify more with the realities of young people today than older staff with retrospective views [42]. Following this principle, Goodwin [29] reports that more than 50% of staff in their MHSU service, which they intend to be youth friendly, are under 23 years of age.

#### Welcoming staff

The youth friendliness of a MHSU service environment incorporates not only service providers, but also support staff such as receptionists and other collaborating professionals [40]. By being welcoming, offering a smile when appropriate^#,^* and a casual “hello, how are you,” the full range of staff members can help make a service youth friendly [37, 44]. Notably, youth may interact with support staff such as receptionists every time they access services, unlike clinicians whom they only see during particular appointments [44]. The characteristics of these support staff may be a guiding factor in bringing youth back into services and should therefore have youth-friendly demeanors [38, 44]^#^.

#### Service provider values & attitudes

A number of personal characteristics, or virtues, were considered important in making a service provider youth friendly. Notably, Hyman et al. [30] found that to be youth friendly, service provider should be “active listeners,” have “positive personality traits” (i.e., friendly, nice, patient), and be “understanding.” While many virtues were listed across the full range of documents reviewed, several emerged as the most dominant: being nonjudgmental [30, 38, 44], respectful [35, 38, 44], genuine [35, 38, 44], honest/direct [30, 35, 44], and “cool” or similar to youth [30, 35, 44]. In stakeholder consultations, service providers notably agreed that genuineness was important since youth can identify when staff are not genuine. They highlighted the importance of using creativity when working with youth and being curious about the youth experience^†^. However, they also emphasized that it would be difficult for one service provider to embody all of the values and aspects of youth friendliness, citing concerns around personal limitations, work-life balance, and burnout. Inter-professional support, possibly including peer support workers for staff, may help staff be more youth friendly^†^.

#### Communication and counselling skills

Youth appreciate when staff have an easy and informal communication style [44], and are dressed casually^†^. Youth recommend that staff communicate in a manner appropriate for a youth’s age, introduce themselves and explain their role [25]. Staff should have knowledge about slang used by youth, which may change rapidly^†^, and clarify when the meaning is unclear [32, 34]. Using humor helps to reduce a youth’s anxiety and achieve an open and relaxed rapport [32, 34, 40], but should be used situationally^†^. Service providers should build trust, with mutual understanding, so youth do not feel pressured to divulge information [40]. They are also encouraged to use a trauma-informed lens [49].

A youth-friendly service provider genuinely* listens to youth and validates their feelings and thoughts [44]. Youth prefer an open session atmosphere where they can talk about anything, including current worries and issues other than the main issue, without interruption [40]. When staff remember the reasons a youth came to the service, it makes the service more youth friendly [44]. Repeating their stories can be discouraging and may reduce the impact of treatment [39].

Staff also recognize that youth may not know about mental health services; guiding youth in navigating services is therefore helpful [28]. Staff should discuss youth’s expectation regarding therapy and clarify any misconceptions [32, 34]. Likewise, staff may have to address their own misconceptions, to ensure they understand what a youth is communicating.* Youth also appreciate receiving practical advice [32, 34] and clear guidelines about how to solve problems [40]. In addition, they appreciate appropriate information about how treatment is expected to help [40].

Self-disclosure can help staff communicate with youth who find it difficult to talk about personal matters directly, e.g., when staff provide information about themselves, it may encourage youth to tell their own stories [32, 34]. Counsellors need to use sound clinical judgment and base their communication on what is most beneficial for youth^†^; this is particularly important for youth who may find it difficult to maintain boundaries [32].

### Treatment/service characteristics

The final overarching category that has been suggested to make MHSU services youth friendly is the treatment or service itself. A number of characteristics were found to make a treatment/service more youth friendly.

#### Location and access

Youth are concerned about the location of services and how to access them, i.e., using public transport or having their parents drive them [40]. MHSU services need to be located somewhere that youth can easily find and access [39, 49], such as close to public transit or in places such as shopping centers or youth centers [40, 46]^†^. Location can be associated with stigma, which may prevent youth from accessing services [39]. For example some youth appreciate a discreetly located service to avoid being seen, whereas others prefer a location closer to other services, such as physical health care, since they can be perceived as accessing the other, less stigmatized service [38]. Although some youth may consider school-based MHSU services stigmatizing if their peers are aware of their service use [24], school-based services may also be useful for some youth^#^.

#### Appointment and wait times

A number of documents suggest establishing flexible appointment times that are convenient for youth needs and schedules [32, 38, 40, 46, 49], e.g., outside school hours [38, 40], work, or other daytime activities. Sessions should be long enough for youth to avoid feeling rushed [40], or flexible, depending on each youth’s needs (e.g., youth may just want to drop in to have a chat) [32, 34]. Drop-in visits and telephone consultations may also be helpful [40]. For example, Goodwin’s [29] program provided a free phone number for consultations. Using text messages to provide appointment reminders may prevent youth from missing appointments [35, 38]. Youth may respond to text messages much sooner than other communication approaches^†^. If privacy and confidentiality is ensured, text messaging can be used to communicate with youth more effectively^†^.

Minimal wait times are essential, since a youth’s life situation may change rapidly [39]. With long wait times, youth may age out of services, or their MHSU situations may deteriorate, potentially opening the door to catastrophic outcomes such as suicide attempts*^,#^. Immediate crisis management services are essential^#^. Some have suggested the benefits of providing an ‘access worker’ to support youth during wait times when waits are unavoidable [38]. Such a support worker could check in with the youth during the wait*, provide support during critical times and increase the youth friendliness of the service [38].

#### Cost

Youth who cannot afford services will not likely access them*. Thus, youth-friendly MHSU services are affordable, free or inexpensive [29, 38, 46].

#### Youth-practitioner fit

A service should have service providers available representing locally prevalent diversity factors, e.g., gender/sexual diversity, cultural diversity.^#^ For example, a female youth with history of abuse may prefer talking to a female counsellor [38]. Youth should therefore be asked whether they prefer a particular service provider or certain provider characteristics^#^. Youth may prefer a consistent service provider, since it is frustrating to lose access to a worker with whom they have established rapport [38, 39]. It may therefore be important to tell youth when a given service provider is available, and having that worker occasionally check in with the youth (e.g., a phone call or a text message), even if they cannot offer optimal appointment flexibility^†^. If rapport is not established, or rapport breaks down, urgent recognition and transition to another appropriate service provider in a timely manner may be necessary [35].

#### First contact and assessment

A prolonged assessment process during the first contact may discourage youth and affect retention [39], especially after a protracted wait time^#^. An informal first contact, without a long paperwork process, is considered more youth friendly [39]. Consistent with an organizational commitment to using technology, using electronic means of assessment (e.g. iPads) may be more youth friendly^#^. Goodwin [29] suggests a simple assessment process using the CHEADS framework (Culture, Home, Education, Activities, Drugs, Alcohol, Sex, Suicide), which utilizes a holistic, developmental and strengths-based framework. Alternatively, an initial session may be offered to establish rapport with youth and provide initial counselling, followed by a detailed assessment during follow up sessions^#^.

#### Youth voice: youth involvement in treatment decisions

Youth need to be well informed about their care, so that they are able to ask questions and make decisions about it [38]. They may be presented with all of the available treatment options, and may be asked if they know about a treatment option that staff could potentially provide.^†^ It is encouraged to solicit youth feedback after every session, to ask what they appreciated and what might not have worked for them^†^. For youth who may be apprehensive about sharing their feedback, an online, anonymous feedback form may be made available^#^. Informed decisions and feedback that is then reflected in the care plan may help build rapport and give youth control over their care [38].

#### Individualized response

It is essential to provide an individualized response [35, 44], since every youth is unique^#,†,^*. This may require flexibility by service providers, i.e., regarding the time it takes to establish rapport, the topic and pace of conversation, and duration of interaction with the youth.^†^ For example, some youth may prefer a strengths-based framework that helps youth build resiliency and resourcefulness^†^. Individualizing services requires building an understanding the youth’s personality and matching services to the youth^#^. Ensuring genuine follow up with every youth after services can help determine satisfaction or identify any need for ongoing services^#^.

#### Recreational approaches

Physical or recreational activities may complement youth services [32, 34, 39]. “Right here” follows the evidence-based approach of using exercise to promote mental health [39]. Since youth are attracted to activities associated with their interests and hobbies, “Right here” offers physical activities rooted within mental health promotion. Staff may be encouraged to engage with youth in activities like playing in the school yard or gym, hiking, fishing, or working on a project together [32, 34]. Youth may be offered variation within the session, such as a 10 min break for a fun activity [40]. Recreational activities help establish rapport and facilitate exploration of sensitive issues [32, 34]. Activities should be developmentally appropriate [40].

#### Group therapy and group activities

Group therapy or support activities may be helpful for some youth, since this format gives them the opportunity to meet other youth with similar experiences^†^. When conducting group therapy, it is important to develop group agreements that encourage all attendees to respect each other’s boundaries and diverse cultures/identities [49]. Group activities may involve structured groups^†^, drop-in groups^†^, or groups with recreational activities embedded in them [32, 34].

#### Artistic and innovative approaches

Some youth appreciate innovative approaches or using non-verbal ways of communicating, e.g. drawing or writing about their emotions [40]. Art therapy, drama therapy or music therapy may be consistent with youth-friendly services [34]. An example of an innovative way of interacting with male youth is using superhero metaphors in treatment, which may provide youth with a language and context to talk about trauma-related experiences [43]. However, some youth may not appreciate using a superhero metaphor, since not everybody has to be a “superhero”*.

#### Caregiver involvement

Youth may attend services on the insistence of their caregivers rather than in a self-motivated manner [40]. While this may promote service access, youth have reported that an adult voice may subdue their own voice during the service process [40]. However, caregivers want to be involved in decisions and informed of the youth's condition^#^. Given the support that caregivers can provide, the caregivers we consulted suggested that youth who do not have a caregiver may benefit from having a supportive worker assigned to them.^#^ In addition, service providers suggested that isolated youth may be encouraged to develop a network of informal supporters for situations in which staff are unavailable, e.g., outside working hours.^†^

### Expected impact of youth-friendly mental health and substance use services

A number of papers described the expected impacts of making MHSU services more youth-friendly in terms of treatment outcomes, although empirical evidence backing these impacts is lacking. Nevertheless, it is hypothesized that service uptake and retention would be improved by implementing many of the youth-friendliness recommendations, including youth engagement in multiple aspects of the organization [38], service promotion [39], youth-friendly environment characteristics [24, 38], youth-friendly staff and service providers [30, 32, 34, 35], and hearing the youth voice at the service/treatment level [38].

As a cornerstone to youth friendliness, integrating the youth voice in planning and delivering MHSU services is expected to increase service uptake and improve outcomes by making the services more acceptable to youth [39]. Youth engagement is expected to lead to reduced stigma, stress, and suicidality, combined with increased coping, empowerment, organizational transparency, and connections among youth, while supporting youth in becoming future professionals in the field [23, 39]. In a bidirectional manner, if an organization establishes youth-friendly characteristics such as flexible times, listening to youth opinions, and applying their ideas, this may increase youth engagement in the organization [47], which is expected to translate into the improved youth friendliness of the organization. Authentic youth-friendly MHSU services may lead to a social/system level change, and improve health equity^†^. In the long term, by improving youth’s social situations^†^ and outcomes, more youth-friendly services may reduce the overall cost of services*^,#^.

### Definition

One of our aims of this scoping review was to develop a comprehensive definition of youth-friendly MHSU services to guide future work in this area. Several papers [38, 44, 48] referred to the definition of youth-friendly services provided by the WHO [15], and one [39] mentioned the WHO ‘You’re Welcome’ criteria [51] of youth-friendly services as a definition. A number of other documents alluded to their own definitions of youth-friendly services (Table 3). However, there was no single, comprehensive definition specific to MHSU services. Based on the diversity of the literature and stakeholder feedback, we propose the following definition of youth-friendly MHSU services:

*“A youth-friendly mental health and substance use service is one that is accessible, appealing, flexible, confidential and integrated, where youth feel respected, valued, and welcome to express themselves authentically, without discrimination of any kind; it is a developmentally and culturally appropriate service that mandates youth participation in service design and delivery, to empower youth and help them gain control over their lives.”*

## Discussion

This scoping review examined the literature and obtained feedback from various stakeholders regarding the concept of ‘youth friendliness’ in MHSU services. The characteristics of youth-friendly MHSU services were found to fall into four main categories: organization and policy characteristics, environment characteristics, service provider characteristics, and treatment/service characteristics. The youth voice was found to be a core value across all the four categories. Youth should be engaged in the services from the planning and developing stage through to the implementation and delivery of services [23, 29, 44]. In addition, they should be engaged in designing the environment [49], and be engaged as staff members [42]. It is critical to prevent tokenistic youth engagement and ensure their meaningful involvement, where their feedback is incorporated in the services [47, 52]. In addition to youth engagement, the youth voice should be heard at the service level; i.e., youth should have a say in the services provided to them [38] and service providers should be relatable to youth.

MHSU service organizations should be integrated with a variety of other services, including physical health services, social and vocational services [38, 44]; confidentiality should be incorporated in their policies [38, 39, 49]; services should be promoted using technological platforms [39, 50]; organizations should provide a ‘brave space’ for youth while ensuring smooth transition across services for different developmental stages [24]; they should provide inclusive services [29, 44] that use non-stigmatizing terminology [24, 49]. The environment should have a colorful and appealing décor, with comfortable furniture and informational material [37, 38, 40, 44]. Youth-friendly service providers should challenge pre-existing paradigms [29], welcome youth [44], communicate easily and informally [32, 44], and use a variety of ways to establish rapport with youth [32]. The service is youth friendly when it is accessible [40], affordable [38], flexible in timing and duration [38, 40, 49], individualized [35, 44], and uses innovative treatment and service options that attract youth and keep them using services [34, 40]. Such youth-friendly services are expected not only to improve service seeking by youth, but also to increase their engagement in and satisfaction with the service [38, 39]. Finally, we have proposed a comprehensive definition of youth-friendly MHSU services, based on the results of this scoping review and feedback obtained from the stakeholders.

While this review considered the characteristics that make a service friendly to youth, many of the characteristics are not specific to youth. For example, involving service users in the design and delivery of services [53] and providing affordable treatment [54] that is acceptable and appropriate [55] may apply to adult populations as well. On the other hand, many characteristics were identified that are specific to youth; for example using youth-appropriate décor, employing technology for assessments, and having knowledge of rapidly changing youth culture and youth language are aspects that may apply differentially to a youth population. Nevertheless, all of the suggestions, whether specific to youth or encompassing a broader age range including youth, should be considered when developing a service in the most youth-friendly manner. As a transitional age group bridging the gap between childhood and adulthood, youth’s service setting preferences are not mutually exclusive from the needs of other age groups, but overarching and comprehensive, with youth-specific components. Engaging youth in all aspects of the organization is key to ensuring that the services are developed in a way appropriate to youth, helping to incorporate not only aspects that might be considered universally “friendly” across the age ranges, but aspects that are specific to a young population and sensitive to rapid cultural shifts as new generations of youth enter services.

It is important to consider the importance of integrated services that span the transition from child services to adult services, which is often abrupt and without youth-specific transitional-age services tailored to this developmental stage [56]. Thus, having services that span the age range of 12–25 years and are tailored to a diversity of youth needs can help to make the transition process smoother [57]. This aligns with the international movement toward integrated youth MHSU and wellness centers [58, 59]. Of course, youth are not a homogenous group, and different youth may have different preferences. It may be necessary to elicit youth feedback about services to orient the services to the local youth population. Likewise, it is important to consider balance between the professional nature and the youth-friendliness of a MHSU service; i.e., if the focus is excessively on youth-oriented aspects, the professional goal and purpose of the service could be forgotten and some youth may find it too “youthy” [38, 44].

This review revealed an important contradiction with regards to confidentiality. On the one hand, youth do not wish to be required to retell their stories [38, 39], and caregivers wish to be informed of their youth’s situations^#^. On the other hand, youth want confidentiality [29, 38, 39, 49], which may conflict with the open communication required to prevent them from having to retell their stories and to meet the needs of concerned caregivers. It is important for MHSU service agencies to take this dichotomy into account and to address consent and confidentiality issues in a way that maximizes the seamlessness of inter-professional collaboration, involves caregivers at the appropriate level, and respects youth’s wishes and rights with regard to confidentiality [60]. They may consider discussing the complexity of confidentiality directly with the youth, including the impacts of sharing their story with their caregivers and other service providers, to help the youth come to an informed decision to guide information sharing. Caregivers may be provided support in the form of caregiver-specific services (e.g., [61]), which may help meet their needs while maintaining the confidentiality of youth.

Some aspects of youth-friendliness in MHSU services overlap with the WHO guidelines for youth-friendly health services as a whole [15]. For example, WHO also mentions that services should be promoted in the community, be inclusive to all youth, be free or affordable, with convenient appointment times and location, confidential, appealing, and have non-judgmental and relatable service providers. However, other aspects of the findings distinguish MHSU service settings from the overarching principles identified by WHO. These include the importance of non-stigmatizing terminology specifically regarding MHSU services; establishing a ‘brave space’; having peer support workers; hiring young staff to whom youth can relate; conducting youth-friendly MHSU assessments using electronic devices (e.g. iPads); individualizing services depending upon the needs and preferences of youth; and providing diverse, innovative service options from which youth can choose. These differences highlight the importance of considering youth friendliness specific to MHSU services.

These findings open the door to a range of future research opportunities. Notably, there is considerable heterogeneity among youth populations, and a single set of youth-friendliness guidelines are not expected to satisfy all youth; future research should consider culture and subgroup differences in youth preferences and ways in which these can be accounted for feasibly in MHSU services. In addition, no papers were found that explicitly studied the association between youth-friendly MHSU services and treatment outcomes. Researchers are encouraged to use creative qualitative and quantitative methodologies to determine the impacts of enhancing the youth friendliness of MHSU agencies. For example, future research should examine the mechanisms and impacts of youth friendliness – if, how, and why it impacts youth service utilization and outcomes. To make this possible, a psychometric tool to assess the degree of youth friendliness of a given agency should be developed to transform the concept of youth friendliness into a measurable construct.

### Limitations

This scoping review has several limitations. The number of stakeholders consulted in our study was sufficient to contextualize the findings of the scoping review, but a larger sample size in a stand-alone qualitative study would support greater generalizability, particularly regarding youth consultations. In addition, only documents available in the English language were selected for this review; however, it has been found that the English-language focus does not create systematic biases in review papers [62]. The search terms were narrow and specific to ‘youth friendliness’ or ‘youth welcoming’. This may be a limitation, but alternately may be considered a strength since it enabled us to define youth-friendly MHSU service, which, to the best of our knowledge, has not been comprehensively defined before. Although a number of articles reported on youth perspectives, it is important to note that this data was analyzed by adult researchers and was therefore filtered by an adult lens; the actual findings may therefore contain an adult bias. However, by abiding by the key finding that youth should be directly engaged in all aspects of an agency, youth-serving organizations can ensure that they take their guidance directly from their own representative group of youth, employing the current findings as a guideline to stimulate discussion with their youth representatives. It should be noted that the grey literature was representative rather than exhaustive. In addition, following the principles of the scoping review methodology [17], an assessment of the quality of studies was not conducted in our review. However, by describing the type of document in Table 3, we have allowed some comparison of studies; in addition, we have strengthened by soliciting stakeholder feedback, which adds methodological rigor [20].

## Conclusion

This scoping review provides a comprehensive overview of the components of youth friendliness that MHSU organizations can use to enhance the youth friendliness of their services. A variety of steps can be taken to make MHSU services more youth friendly, potentially increasing service seeking, service uptake, and satisfaction. Integrating the youth voice into MHSU services—in the form of youth engagement at all levels of an organization—was found to be a core component of youth-friendly services. Further research is required to measure the impact of youth-friendly services on service outcomes.

## Additional file

Additional file 1: Youth Friendliness Scoping Review Search Strategy – MEDLINE Search. The comprehensive MEDLINE search strategy is detailed in this file (DOCX 19 kb)

## Abbreviations

LGBTQ: Lesbian, Gay, Bisexual, Transgender, Queer
MHSU: Mental Health and Substance Use
WHO: World Health Organization
