# Supplementary material for: What makes mental health and substance use services youth friendly? A scoping review of literature
Source: BMC Health Serv Res. 2019 Apr 27;19:257. doi: 10.1186/s12913-019-4066-5 (PMC6486969; doi:10.1186/s12913-019-4066-5)
Supplement: Supplementary file 1 — Youth Friendliness Scoping Review Search Strategy – MEDLINE Search. The comprehensive MEDLINE search strategy is detailed in this file (DOCX 19 kb) [file 12913_2019_4066_MOESM1_ESM.docx]

**Youth Friendliness Scoping Review Search Strategy – MEDLINE Search**

The comprehensive MEDLINE search strategy is detailed below. This strategy was adapted to the other databases searched.

**Database: OVID MEDLINE Epub Ahead of Print, In-Process & Other Non-Indexed Citations, Ovid MEDLINE(R) Daily and Ovid MEDLINE(R) 1946 to Present**

--------------------------------------------------------------------------------

1 Mental Health/ (28165)

2 mental health.ti,kf. (49938)

3 exp Mental Health Services/ (84949)

4 exp mental disorders/ (1076152)

5 ((behavio?r* or mental) adj2 (health or disorder* or problem*)).ti,kf. (83459)

6 psychiatry/ or adolescent psychiatry/ or child psychiatry/ or psychology, child/ or psychology, developmental/ (55222)

7 ((obsessive compulsive or panic or post traumatic stress or eating or bipolar or oppositional defiant or conduct) adj2 disorder*).ti,kf,kw. (39266)

8 (depress* or anxiety or substance abuse or drug abuse or alcoholism or alcohol abuse or disruptive behavio?r disorder* or attention deficit or autism or asperger*).ti,kf,kw. (228156)

9 (agoraphobia or PTSD or trauma or OCD or anorexia or bulimia or psychosis).ti,kf,kw. (101970)

10 or/1-9 (1360157)

11 ((youth or adolescent* or teen* or young adult* or young people or emerging adult*) adj friendly).tw. (401)

12 ((youth or adolescent* or teen* or young adult* or young people or emerging adult*) adj2 welcoming).tw. (2)

13 Adolescent Health Services/ (4970)

14 (friendly or welcoming).tw. (25903)

15 13 and 14 (131)

16 11 or 12 or 15 (425)

17 10 and 16 (87)

18 exp africa/ or exp caribbean region/ or exp central america/ or exp "gulf of mexico"/ or latin america/ or mexico/ or exp south america/ or exp asia/ (1056598)

19 17 not 18 (66)

20 limit 19 to (comment or editorial or letter) (1)

21 19 not 20 (65)

22 limit 21 to english language (62)

23 limit 22 to yr="2001 -Current" (61)

24 remove duplicates from 23 (58)

*************************
